# A Study of Perceived Nature, Shade and Trees and Self-Reported Physical Activity in Denver

**DOI:** 10.3390/ijerph16193604

**Published:** 2019-09-26

**Authors:** Sara Tabatabaie, Jill S. Litt, Amanda Carrico

**Affiliations:** Environmental Studies Program, University of Colorado Boulder, Boulder, CO 80309, USA; sara.tabatabaie@colorado.edu (S.T.); Amanda.Carrico@colorado.edu (A.C.)

**Keywords:** physical activity, perceived greenness, shade, trees, objective greenness

## Abstract

(1) Background: Current evidence on the association between greenery and physical activity (PA) remains inconsistent. Most studies on this association use objective measures of greenness, which do not reveal people’s perceptions of greenness in neighborhoods, or the role of quality components of greenness, such as shade, trees, and the presence of nature on this association. (2) Methods: Drawing on data from the Neighborhood Environment and Health Survey—a cross-sectional population-based survey of Denver residents in 2007—we examined which measures of greenness (perceived and objective) correlated with the self-reported PA. We also assessed how components of perceived greenness, shade, trees and the presence of nature, correlated with PA. (3) Results: Perceived greenness, reflecting perception of trees, shade and the presence of nature, was positively associated with reported moderate–vigorous PA. Conclusion: Findings provide evidence that quality aspects of greenness affect people’s perception of the neighborhood in a way that relates to PA. The individual contributions of shade, trees, and the presence of nature in this association should be analyzed in future studies. Understanding the link between shade and trees and PA has implications for how to plan for walkability and sun safety at the neighborhood scale.

## 1. Introduction

Integrating physical activity (PA) into daily routines has become a national priority following the American College of Sports Medicine/Centers for Disease Control’s statement on PA and health [1] and the U.S. Surgeon General’s Report on Physical Activity and Health [2]. The 21st-century active-living agenda is concerned with the negative health impacts of physical inactivity and poor diet [3]. This agenda calls for strategies that target multiple levels of influence (individual, inter-personal, organizational, community and policy) to promote PA. Moderate-intensity activities, such as walking, are deemed an important way to achieve daily PA goals. Neighborhood-based strategies offer opportunities to make moderate-intensity activities such as walking and biking a possibility for people living in all kinds of neighborhoods and across all kinds of social groups. 

Neighborhood design can have direct and indirect effects on PA and people’s perception of the desirability of the neighborhood for PA, respectively [4,5,6]. Active-living research priorities include understanding streetscape features within the neighborhood context and their role in shaping PA levels. As a component of neighborhood environments, urban greenness—urban parks, and street greenery in particular—seems to advance PA [7,8,9,10,11,12,13,14]; however, evidence on the link between urban greenness and PA remains inconsistent due to measurement variations, definitions, context and cultural differences, and small sample sizes [15,16,17,18,19,20,21].

### 1.1. Conceptualizing Perceived Greenness through Components of Landscape Quality: Trees, Natural Features, and Shade

Neighborhood greenness has been shown to foster positive perceptions of the neighborhood environment and, therefore, influences a variety of pro-health behaviors and improved health status [7,8,9,10,11,12,13,14]. The “quality” of landscapes and vegetation is a facet through which people experience the place, perceive the desirability of the area and interact with the surrounding environment. Gobster (2007) posits that a human’s response to the environment is affected by his/her experience of the surrounding landscape. He defines the “perceptible realm” as the scale at which humans perceive the environment. We perceive the surrounding landscape through all our senses. He suggests that people tend to interact with or protect landscapes that are perceived as more aesthetically pleasing [22]. Moreover, studies suggest the quality of landscape is a facet through which the surrounding environment may affect health behavior [23,24].

The quality of greenness is closely related to perceived neighborhood aesthetics, which has been parameterized in different public health and planning studies. For example, Pikora et al. (2003) assesses streetscape aesthetics using eight items: cleanliness, sights, garden maintenance, parks, pollution, trees, architecture, and street maintenance [25]. For Ball et al. (2001), neighborhood aesthetics are defined by the level of friendliness of the neighborhood, the attractiveness of the local area, and the invitingness of the neighborhood for walking [26]. Handy et al. (2002) measured neighborhood aesthetics based on the percent of ground in shade at noon, and the lack of incivilities (number of locations with graffiti per square mile) [27]. For Sugiyama et al. (2010), the aesthetic factors that made areas more conducive to recreational walking include the suitability of the area for chatting and children’s play, the welcoming and relaxing quality of the area, and the quality of trees and plants [28]. Saelens and others (2003) assessed neighborhood aesthetics according to the existence of trees and natural sights, adequate shade, attractive landscaping, and attractive buildings in the neighborhood [23,29]. Most of these studies have shown a positive relationship between the aesthetic quality of streetscapes and PA [8,25,26,30].

The presence of trees and natural features are common neighborhood characteristics that represent the quality of greenness and have been linked with PA behavior [16,17,31,32,33]. Shade is another facet of the quality of the natural environment but has mostly been studied within the parks and educational settings [34,35,36,37,38,39]. However, shade should be included in studies of nearby nature and PA, given that outdoor-based PA may carry some risks due to extreme heat, thermal discomfort [40], and harmful levels of UV exposure from the sun. Shade is viewed as a precious commodity to reduce the hazards of excessive UV exposure and rising temperatures and is created naturally by tree canopies or artificially through shade structures. It also contributes to the aesthetic quality of streetscapes and can affect people’s PA behavior [25,41,42,43].

### 1.2. Measuring Quantity of Greenness through Objective Assessments of the Environment

Neighborhood greenness has been measured differently in studies that link PA to greenness. The three main ways greenness is assessed include perceived measures of greenness using surveys, observed measures of greenness using valid and reliable audit tools, and objective measures of greenness derived from GIS and satellite imagery. According to Leslie (2010), a majority of studies that show significant relationships between greenness and PA are those that collect perceived greenness measures (e.g., perception of trees, shade, and the presence of nature). In contrast, studies that use objective measures, such as Normalized Difference Vegetation Index (NDVI) or GIS-driven measures, report less significant or no association between greenness and PA levels [28]. Sarkar attributes the inconsistency in the association between greenery and PA to differences in definitions of green space, and different methods applied to parameterize greenery [16].

### 1.3. Measuring Quality of Greenness through Perceived Assessments of the Environment

While objective measures of greenness reflect the quantity of greenery or access to green space, perceived measures reveal how people perceive the presence or quality of greenness. Some studies suggest that the size and distance to green space do not predict the effective use of the green space, but there are other factors that may affect the use of urban greenness [44]. Also, in some instances, a large green space in urban areas may increase distances between other amenities and destinations resulting in less walking or cycling [45].

### 1.4. Factors Mediating People’s Perception of the Environment

People’s perception of the surrounding environment and the way this perception affects health behaviors, PA behavior in particular, may be influenced by their attitudes and socio-economic characteristics, such as age, ethnicity, and level of income and education [46,47]. People’s perception of safety has been shown to affect PA behavior in many studies [48,49,50,51]. The time of the year may also affect this perception. Some studies also show that the level of the walkability of the neighborhood may affect its residences’ perception of the surrounding areas [52]. Moreover, the length of residency in the neighborhood is important because it can influence people’s relationships to the neighborhood environment and related perceptions of this place [53,54]. These factors are considered important mediators of the association between greenness and PA.

### 1.5. Exploring Associations between the Quantity and Quality of Greenness and PA Behavior

Although there is evidence suggestive of a link between neighborhood greenness and PA behavior, there are still discussions over the importance of the quantity versus the quality of greenness in this association. In this study, we aim to assess the following: (1) which measures of greenness—perceived and/or objective—are correlated with reported PA at the block group level, one type of proxy for neighborhoods; (2) whether and how this association differs for moderate–vigorous, and light levels of PA; (3) how the quality of perceived greenness, including available shade, trees, and the presence of nature, correlates with PA. We draw on data from the Neighborhood Environment and Health Survey, a population-based survey [55], to explore the relationships between perceived and objective greenness and reported PA. Specifically, we hypothesize that PA is more significantly correlated with perceived greenness, as measured by perceptions of the presence of trees, shade, and nearby nature than the quantity of greenness as measured by NDVI. We also hypothesize that positive perceptions of greenness influence all types of PA behavior, including light, moderate and vigorous levels of PA.

## 2. Materials and Methods

Data for this study were collected using a population-based survey and neighborhood audits across 92 block groups in Denver, Colorado. (Census block groups are geographic units, between census blocks and census tracts that contain between 600 to 3000 people. Block groups are used by the United States Census Bureau to present census data.) The survey was conducted from 2006 to 2007. Block groups were located east of a major interstate highway (I-25). A total of 474 residents participated in a 45-minute face-to-face survey. The participants were initially selected using a multi-frame sampling design. This sampling consisted of an area-based sample of the general population and a list-based census of community gardeners with a recruitment goal of 480 households from 40 block groups. A total of 1151 households were randomly selected within the area-based sample. Of the initial 1451 selected households, 648 could not be contacted. Of the 803 contacted households, 474 (response rate = 59%) participated in the survey [56]. One eligible respondent aged 18 or over was randomly selected from each household using the “most recent birthday” method. The sample size was initially determined to satisfy the statistical power of 0.8. The sample size was increased by 45% to account for possible non-respondents. We limited our sample to block groups that included at least two households (*n* = 58) to keep the level of accuracy of the regression coefficients and variances [57,58]. The average sample size was 7.5 participants per block group. More details about sampling and study design can be found in the following sources [14,55,56].

The survey questionnaire was developed based on existing surveys on neighborhood and health and included items on aesthetics [29], social involvement [59], neighborhood attachment [60], physical activity, and dimensions of health [14,55,56]. Questions were available in English and Spanish. Data were collected through a 45-minute face-to-face survey at or near the home of the respondent.

In support of this analysis, we developed one objective measure of the neighborhood greenness: the Normalized Difference Vegetation Index (NDVI) for each block group. NDVI for each block group was created using Environment for Visualizing Images (ENVI) software. Landsat images acquired from USGS Earth Explorer were used to develop NDVI, which is an index of photosynthetic activity or plant greenness. NDVI shows the density of green on a patch of land by calculating the normalized difference between the red and near-infrared bands from an image. A larger NDVI value corresponds to denser vegetation.

$$\mathrm{NDVI}=\;\frac{\left(\mathrm{NIR}-\mathrm{RED}\right)}{\left(\mathrm{NIR}+\mathrm{RED}\right)}$$

The reason that we selected NDVI as the objective greenness variables was because most studies evaluating the association between objective greenness and PA have used NDVI as the measure of greenness. Using this measure allows us to compare our results with similar studies. NDVI was attached to the dataset via the block group ID.

*Dependent measure: Self-reported physical activity*

We used the Community Healthy Activities Model Program for Seniors (CHAMPS) Physical Activity Questionnaire to assess PA behavior. This instrument captures the weekly frequency and intensity of undertaken activities, such as swimming, tennis, golf, dance, walking for the purpose of leisure or exercise, and light and heavy gardening. Outcome measures denoted the weekly frequency and duration of PA undertaken by adults at light (e.g., leisurely walking, light gardening), moderate-vigorous (e.g., cycling, heavy housework, tennis and jogging) and total hours of PA/week [61]. We generated two measures of PA, moderate-vigorous, and light, by combining the reported hours of PA items according their metabolic equivalent value (MET value). The moderate-vigorous category includes physical activities of MET ≥ 3 [61,62].

*Composite variable: Perceived green*

Participants’ perceptions of trees, shade and the presence of nature in the neighborhood were measured in the survey. Respondents rated these features on a scale from 1 (Strongly Disagree) to 4 (Strongly Agree). These three variables showed high correlations (r > 0.4); we performed an Exploratory Factor Analysis (EFA) to account for high correlations between these variables and explore the latent variables. The factor analysis resulted in one factor, namely perceived green. This composite variable evaluates people’s perceptions of trees, shade and the presence of nature while they are walking in the neighborhood (α=0.76) (Table 1).

*Composite variable: Perceived safety*

Participants’ perceptions of being attacked or robbed, and overall crime in the neighborhood were measured in the survey. Perceived safety is a composite variable, assessing people’s fear of crime and their idea about safety in their neighborhood (α=0.85) Table 2.

*Objective Greenness*

As described above, we used NDVI as the objective measures of greenness.

*Control Variables*

We included self-reported age category, gender, level of education (1 = not high school graduate; 2 = high school graduate or some college; 3 = college grad), the presence of children under 18 in the household (yes/no), and ethnicity dummy variables (White, African American, and Hispanic) as socio-demographic control variables [46]. Self-reported health was included as a proxy for health status. This item was assessed using a single item from the Behavioral Risk Factor Surveillance System Survey (BRFSS), showing how respondents rate their general health on a scale of 1 (poor) to 5 (excellent). It has been shown to be a valid and reliable measure of health status [63].

*Walkability Index*

We used the GIS-derived macro-level walkability index (WI) to classify our block groups into low and high walkable areas. The walkability index was derived as a function of net residential density, intersection density, retail floor area ratio, and land use mix [64,65]. We applied GIS to develop the WI. Data were retrieved from the City of Denver Open Data Catalog. The mean WI was used as the break point between low and high walkable areas.

*Seasonal effect*

Through our analysis, we found an association between the month of the interviews and reported PA levels. The maximum level of PA was reported in June and July. Reported hours of PA showed a linear decrease before and after these two months. We recoded the month of interview to reflect this pattern and used this variable in our models to adjust for seasonal effects. In the new scheme, we defined two intervals for the first and second halves of the year. In the first interval, our variable increased from Jan = 1 to June = 6; in the second interval, our variable decreased from July = 6 to Dec = 1.

*Length of residency*

The length of residency variable represented the number of years respondents lived in the neighborhood (1 = 1–2 yrs; 2 = 2–7 yrs; 3 = 8–17 yrs; 4 = >17 yrs).

*Data analysis*

We applied multilevel statistical models to examine the association between two levels of PA and greenness measures, while accounting for the differences in PA across block groups. We calculated the statistical models of respondents nested in block groups via PROC MIXED in SAS version 4.9. This software is manufactured by SAS Institute Inc. based in Cary, North Carolina, USA.

We applied the random intercept model to develop the block group models. The multicollinearity between variables was checked using variance inflation factors (VIF). We did not find significant problems with multicollinearities (VIFs < 2.5). We started with intercept-only models to understand the variation of PA across the block groups. We then developed our preliminary multivariable models by adding the control variables—age, educational attainment, gender, ethnicity, self-reported health, the presence of children under 18 in the household, the length of residency in the neighborhood, and the month of the interview—one at a time to the intercept model (data not shown). Of these variables, ethnicity, the presence of children under 18, and the length of residency were excluded from models in the multivariable analysis because they showed non-significant regression coefficients in the univariate analysis.

Greenness variables were added to the preliminary models one at a time. Models with the lowest -2loglikelihood were selected as the best models (Models A–B). The perceived safety variable, as well, was excluded from multivariable models because it showed a non-significant regression coefficient in the univariate analysis.

We also checked whether the level of walkability affected the association between PA and the green variables by adding the walkability index to the models (Models C–D). Prior to this step, we checked the significance of interaction terms (WI x perceived green), (WI x self-reported health), and (WI x NDVI) using PROC GLM. Interaction terms were excluded from models since their coefficients were not significant. Table 4 lists final models, including only statistically significant variables.

## 3. Results

Table 3 summarizes the characteristics of the respondents in our analysis and shows hours of PA by demographic and socioeconomic characteristics. Five records were dropped because of missing values (N = 469).

Participants were mostly women (67.4%), White (57.8%), and obtained a college degree or higher (57.4%). The average age of participants was 46 years. Most respondents reported good, very good, or excellent health (80.3%). On average, respondents did 7.8 h of light PA/week and 10.7 h of moderate-vigorous PA/week.

The reported hours of moderate-vigorous PA were statistically higher among males, Whites, and respondents with at least a college degree. The hours of moderate-vigorous PA were also higher among respondents who reported a better health condition. The hours of light activity were higher among respondents between 65 and 95 years old, as well as among participants who reported a poor health condition.

Table 4 presents the estimated univariate parameters (β) and results from multi-level models (A–D). The reported hours of PA at moderate–vigorous level was positively associated with people’s perception of greenness in their neighborhoods, after adjustment for educational attainment, age, gender, health, and the month of the interview. This association was not significant for PA at light level (Models A and B).

This result implies that components of perceived greenness, participants’ perception of shade, trees, and the presence of nature positively affect levels of PA at moderate–vigorous level. However, a high correlation between the components of the perceived green variables—shade, trees, and the presence of nature—does not allow us to tease out the exact contribution of each of these items in the association between PA variables and the perceived green variable (Table 5).

The results did not show a significant association between the two levels of PA and the objective measure of greenness, NDVI, after adjustment for demographic variables and the month of interview.

Adjusting our models for the objective level of walkability (Models C and D), coefficients of the walkability index were not statistically significant, suggesting that the relationship between PA variables and the perceived measure of greenness did not vary with the objective level of walkability.

## 4. Discussion

The present study aimed to explore the association between quality, and quantity measures of greenness and reported light and moderate–vigorous hours of PA and perceived and objective measures of greenness. It also examined whether mediator factors, such as the level of walkability affects such associations. Moreover, we aimed to investigate how quality components of greenness, trees, shade, and the presence of nature, affected the PA behavior.

Our results suggest that moderate–vigorous PA is positively influenced by the perceived greenness, which reflects people’s perception of quality components of greenness in the neighborhood: trees, shade, and the presence of natural features. On the contrary, we did not observe a significant association between the quantity of greenness, represented by NDVI, and PA. Our analyses also suggest that the association between perceived greenness and PA does not vary by objective walkability levels.

### 4.1. Quality of greenness and PA Behavior

This study supported our hypothesis that there is a significant positive association between perceived greenness and PA behavior. Different mechanisms may explain this association. Quality characteristics of greenness, trees, shade and natural features provide opportunities for mental restoration and stress release [66]. Greenness advances neighborhood attachment by fostering aesthetic experience [67]. Also, street greenness is shown to be a proxy of neighborhood wealth, implying higher levels of maintenance and aesthetic qualities and lower incivilities in the neighborhood [68]. These qualities contribute to a higher perception of environmental comfort, safety and aesthetic, the three main factors that influence the desirability of the environment for PA [6,69].

The implied positive link between shade and PA in our study is consistent with findings from some studies that investigated impacts of shade in public open spaces [70]. Floyd (2008), however, links the level of walking in parks with the lower amount of shade [71]. Kegler’s (2015) findings suggest that people have mixed views on whether shade influences the decisions to exercise in rural neighborhoods [72]. Generally, Camcho (2014) links people´s preferences for trees and greenery to tree/greenery attributes and benefits, most importantly shade and oxygen supply [73]. Several studies suggest that the use of public spaces, such as parks, are influenced by their thermal conditions, indicating the importance of shade in outdoor environments in this regard [40,74,75,76].

### 4.2. Quantity of Greenness and PA Behavior

The lack of association between the quantity of greenness, measured by NDVI, and PA in this study is consistent with other studies [19,21,77]. The mismatch between the perceived and objective greenness measures is not surprising, as these two measures capture different aspects of neighborhood greenness. Perceived measures are usually better at capturing human aesthetic landscape values that derive from experiencing the environment [78], which is affected by individual factors, such as attitude, beliefs, and self-efficacy [79]. NDVI, however, is an indication of plant “greenness” or the quantity of greenery in the area. The perceived greenness, however, included people’s perception of trees, shade and the presence of natural features in the neighborhood; the latter two components are not captured through NDVI. Moreover, NDVI denotes trees, as well as other vegetation, including lawns and groundcover that are both in public realms and private gardens. In reality, people are not exposed to vegetation in private yards while they are out in the neighborhood; therefore, this type of vegetation may not affect their perceptions of the desirability of neighborhood environments for PA [16,28]. Additionally, NDVI reflects the quantity of greenness and does not assess whether people have convenient access to this greenness. Some studies show that between the amount of greenness and access to greenness, the latter has a more positive effect on residents’ PA behavior [80].

### 4.3. Different Types of PA and Positive Perceptions of Greenness

Our study did not support our hypothesis that positive perceptions of greenness have similar impacts on all types of PA. We found a stronger association between the reported moderate-vigorous PA and perceived greenness when compared to the association between reported light PA levels and perceived greenness. This suggests that positive perceptions of greenness many not increase the likelihood of all types of activities, but rather those activities that are undertaken outdoors in the neighborhood environment. Moderate-vigorous PA includes those activities with MET value greater than 3, such as jogging, cycling, walking fast, swimming, and so forth [61,62]. These activities are more likely to be undertaken outdoors in neighborhoods; thus, their occurrence is more likely to be influenced by people’s sense of the place, and their perception of and interpretation with the surrounding environment [6,81]. However, light activities, such as playing instruments, dishwashing, and going to concerts are performed at home or in settings other than neighborhood streets. Thus, the intention of undertaking these “light” activities is less likely to be affected by the perception of the surrounding neighborhood.

### 4.4. The Impact of Walkability

Our findings do not detect a statistically significant difference in the relationship between PA and perceived greenness in high and low walkable neighborhoods. In that, people in both low and high walkable areas showed similar perceptions of the desirability of greenness in their neighborhood for PA. Leslie (2005), however, suggests significant differences in the perception of neighborhood environmental characteristics between residents living in objectively “high” and “low” walkable areas [52]. She found that residents in low walkable neighborhoods reported lower perceptions of residential density, land use diversity and access, connectivity, and walking infrastructure, but higher positive ratings of neighborhood aesthetics. This inconsistency between our results and those of past studies suggests that people’s perception of the desirability of neighborhoods for PA does not always match the objective walkability score. Objective walkability is based on residential density, land use mix, and street connectivity; this factor does not account for design and natural features of the neighborhood that may affect people’s aesthetic perceptions.

There are several limitations concerning this study. First, the CHAMPS instrument does not ask respondents where they have performed PA. In this study, we assumed that a high proportion of the reported hours of weekly activities have been carried out in the vicinity of the respondent’s residence. This assumption might affect the accuracy of the results. Future studies can apply objectively measured, or geocoded PA information to examine the accuracy of our findings. Second, self-reported PA is always over-reported and is subject to social desirability and recall bias [82]. Also, the hours of moderate-vigorous-intensity PA is susceptible to be biased as CHAMPS has been shown to over-report some items, such as walking and housework [83]. Nevertheless, CHAMPS is suggested as a reliable tool to assess moderate to vigorous activities in older adults [83]. Moreover, while the magnitude of PA is over-reported, the proportional differences between social groups and across demographic variables are in the expected directions. Third, as a cross-sectional study, this research cannot make claims about causality between people’s perception of greenness in their neighborhood and PA levels. Moreover, the degree of self-selection bias in this study is unknown and may affect the results. Furthermore, a group of our respondents was recruited from community gardens. Gardeners may have different perception of and interaction with greenness. This fact may affect the accuracy of our results. However, these gardens were located in predominantly low-income and minority areas of Denver, thus providing demographic diversity to our sample. Finally, although the survey data was collected in 2007, the data set is unique in that it allows us to study the relationships between variables of interest, drawing on objective and subjective data across a diverse sample of Denver neighborhoods and Denver residents.

## 5. Conclusions

Our findings suggest that the perception of greenness is important for influencing moderate to vigorous PA among urban residents. Relying solely on the objective measures of the built environment does not fully explain how people interact with the built environment. There are qualitative aspects of greenness, such as shade and landscape aesthetics more generally, that relate to PA. Root et al. (2017) emphasizes the importance of both perceived and objective measures. The authors suggest that the presence and quantity of the greenness by itself may not affect health behaviors, but it is the quality of the green environment and the experience with greenness that facilitates health-promoting processes and, subsequently, health status [23]. One implication is that urban planners and health officials should assess quality aspects of green space in addition to traditional, objective measures such as NDVI, tree canopy, and objective walkability indices. Also, emphasis should be placed on interventions that enhance people’s perception of greenness in their neighborhood.

This research is not conclusive about the attributes of greenness, shade and trees, in particular, that affect the desirability of neighborhoods for PA. Future research studies are encouraged to examine the impact of shade and trees along with other features of streetscapes, such as sidewalks, architecture, landscapes, and maintenance on PA behavior in neighborhoods. Qualitative research methods, such as interviews, participant observation, or a visual landscape assessment survey, can help scholars and planners research the meaning of greenness for streetscapes and to evaluate factors that affect the desirability of streetscapes for PA. Understanding how shade and trees affect PA behavior will contribute to both walkability and sun safety literature.

## Figures and Tables

**Table 1:** Factor loadings and squared multiple correlations of the variables with Perceived Green.

Squared multiple correlations of the variables with the factor	0.73
Factor Loadings	
Perception of the presence of trees in neighborhood	0.78
Perception of the presence of shade in neighborhood	0.74
Perception of access to nature in neighborhood	0.57

**Table 2:** The composite green measure and composite safety measure used in analysis.

Composite Measure	Scale	Cronbach’s Alpha
**Perceived Green**	4-point scale: strongly agree to strongly disagree	0.76
There are trees along the streets in my neighborhood. Trees give shade for the sidewalks in my neighborhood. There are many attractive natural sights in my neighborhood (such as landscaping, views).		
**Perceived Safety** [45] How afraid are you of being attacked or robbed: At home in your house or apartment? On the streets of your neighborhood during the day? Out alone at night in your neighborhood? Out with other people at night in your neighborhood?	4-point scale (very fearful to not fearful)	0.85
Has fear of crime in your neighborhood caused you to limit the places or times that you will: Go shopping? Work? Go by yourself?	Yes/no	

**Table 3:** Number (%) distribution of characteristics of survey respondents by demographic, health, greenness measures and physical activity (PA) variables (N = 469).

Demographic and Health Variables	Characteristics of Respondents Number (%)	Hours of Moderate–Vigorous PA/Week Mean (95% of CI)	Hours of Light PA/Week Mean (95% of CI)
**Demographic and socio-economic characteristics**			
**Gender**			
Male (1)	153 (32.6)	12.2 (10.6, 13.8)	8.1 (7.2, 9.1)
Female (2)	316 (67.4)	10.0 (9.1, 10.9)	7.6 (7.1, 8.2)
**Ethnicity**			
White (1)	271 (57.8)	11.1 (10.1, 12.1)	7.6 (7.0, 8.2)
Non-White (0)	198 (42.2)	10.2 (8.9. 11.5)	8.1 (7.3, 8.9)
**Education**			
Less than high school (1)	52 (11.1)	5.8 (3.9, 7.7)	6.8 (5.4, 8.2)
High school or some college (2)	148 (31.6)	10.3 (8.9, 11.7)	8.1 (7.2, 9.0)
College graduate or higher (3)	269 (57.4)	11.7 (10.6, 12.8)	7.9 (7.2, 8.5)
**Age category**			
18–24 (1)	30 (6.4)	10.0 (7.4, 12.6)	6.7 (4.7, 8.7)
25–44 (2)	195 (41.8)	11.2 (10.1, 12.4)	7.1 (6.5, 7.7)
45–64 (3)	177 (37.9)	11.5 (10.0, 13.0)	7.7 (6.9, 8.5)
65–94 (4)	65 (13.9)	7.2 (5.7, 8.7)	10.8 (9.1, 12.4)
**Children under 18 at home**			
Yes (1)	158 (34.5)	10.6 (9.2, 11.9)	8.3 (7.5, 9.1)
No (2)	300 (65.5)	11.0 (9.9, 12.0)	7.7 (7.1, 8.3)
**Self-reported health**			
Poor	14 (3)	8.9 (4.7, 13.1)	9.9 (5.5, 14.3)
Fair	78 (16.7)	8.4 (6.5, 10.3)	8.0 (6.8, 9.2)
Good	119 (25.5)	9.2 (7.8, 10.5)	7.1 (6.3, 8.0)
Very good	156 (33.4)	10.7 (9.4, 12.0)	7.9 (7.1, 8.8)
Excellent	100 (21.4)	14.2 (12.1, 16.2)	7.8 (6.8, 8.9)

**Table 4:** Multilevel model results on the relationship between PA variables, perceived measure of greenness and individual characteristics.

Independent Variable	Hours of Moderate-Vigorous PA (hr/wk)				Hours of Light PA (hr/wk)			
	Univariate Factor β	Null Model	Model A	Model C	Univariate Factor β	Null Model	Model B	Model D
**Demographic variables**								
Gender (ref: men)	−2.18 **		−1.62	−1.65	−0.55		−0.36	−0.35
Education category (ref: > college degree)								
Less than high school	−5.88 **		−3.84 **	−3.56 **	−1.01		−0.71	−0.79
High school or some college	−1.44		0.17	0.22	0.22		0.34	0.33
Age category (ref: 65 < age < 94)								
18–24	2.79		3.79	3.91	−3.98 **		−3.83 **	−3.85 **
25–44	3.98 **		4.24 **	4.20 **	−3.64 ***		−3.95 ***	−3.93 ***
45–64	4.27 **		4.47 ***	4.42 **	−3.02 ***		−3.36 ***	−3.33 ***
**Health**								
Self-reported health	1.74 ***		1.34 **	1.32 **	−0.05		−0.64	−0.05
**Perceived Variables**								
Perceived green	2.26 **		1.50 **	0.86	0.61		0.66	0.68
**Built environment objective variables **								
NDVI	3.78		−4.02	−2.69	−2.08		−4.53	−4.96
Walkability Index (WI)				0.96				−0.33
**Other variables**								
Month of interview	1.17 ***		1.04 ***	1.04 ***	0.39 **		0.44 **	0.44 **
**Intercept**		10.75 **	−1.22	2.69		7.84 **	10.20 ***	10.40 ***
**Intraclass correlation**			−0.02	−0.02			0.02	0.02
**-2log likelihood**		3069.8	2803.6	2801.9		2839.0	2598.0	2597.0

** *p* < 0.05, *** *p* < 0.0001.

**Table 5:** Spearman correlations between the components of the perceived green variable.

Spearman Correlation Coefficients			
Components of Perceived Green	Trees	Shade	Presence of Nature
Trees	1	0.69 ***	0.48 ***
Shade	0.69 ***	1	0.45 ***
Presence of nature	0.48 ***	0.45 ***	1

*** *p* < 0.0001.
